# Exploring unmet needs and preferences of young adult stroke patients for post-stroke care through PROMs and gender differences

**DOI:** 10.3389/fstro.2024.1386300

**Published:** 2024-05-09

**Authors:** Sarah Ibrahim, Troy Francis, Kathleen A. Sheehan, Kristina Kokorelias, Aleksandra Stanimirovic, Syeda Hashmi, Csilla Kalocsai, Sharon Ng, Suze G. Berkhout, Jill I. Cameron, Valeria Rac, Aleksandra Pikula

**Affiliations:** ^1^Program for Health System and Technology Evaluation, Toronto General Hospital Research Institute, Toronto, ON, Canada; ^2^The Jay and Sari Sonshine Centre for Stroke Prevention and Cerebrovascular Brain Health, Toronto Western Hospital, University Health Network, Toronto, ON, Canada; ^3^Centre for Advancing Collaborative Healthcare and Education, University of Toronto, Toronto, ON, Canada; ^4^Institute of Health Policy, Management and Evaluation, Dalla Lana School of Public Health, University of Toronto, Toronto, ON, Canada; ^5^Department of Psychiatry, University of Toronto, Toronto, ON, Canada; ^6^University Health Network Centre for Mental Health, Toronto, ON, Canada; ^7^Section of Geriatric Medicine, Department of Medicine, Sinai Health and University Health Network, Toronto, ON, Canada; ^8^Department of Occupational Science and Occupational Therapy, University of Toronto, Toronto, ON, Canada; ^9^Temerty Faculty of Medicine, Rehabilitation Sciences Institute, University of Toronto, Toronto, ON, Canada; ^10^Garry Hurvitz Brain Sciences Program, Sunnybrook Health Sciences Centre, Toronto, ON, Canada; ^11^Temerty Faculty of Medicine, University of Toronto, Toronto, ON, Canada; ^12^Harvard Chan School of Public Health, Boston, MA, United States; ^13^Knowledge, Innovation, Talent, Everywhere – Toronto Rehabilitation Institute, University Health Network, Toronto, ON, Canada; ^14^Department of Medicine (Neurology), Temerty Faculty of Medicine, University of Toronto, Toronto, ON, Canada; ^15^Krembil Brain Institute, University Health Network, Toronto, ON, Canada; ^16^Department of Neurology, Toronto Western Hospital, Toronto, ON, Canada

**Keywords:** quality of life (QoL), stroke, young adults, patient-reported outcome measures (PROMs), gender difference

## Abstract

**Background:**

Stroke incidence among young adults of working age (under 65 years of age) has significantly increased in the past decade, with major individual, social, and economic implications. There is a paucity of research exploring the needs of this patient population. This study assessed: (1) young adult stroke patients' physical, psychological, and occupational functioning and health-related quality of life (HRQoL); and (2) post-stroke care preferences using patient-reported outcome measures (PROMs), with attention to gender differences.

**Methods:**

A cross-sectional pilot study was conducted. Sociodemographic and clinical characteristics were collected through chart review and data on occupational function, physical, psychological, and social wellbeing >90 days post-stroke through a self-reported survey. Descriptive statistics, gender-based, and regression analyses were conducted.

**Results:**

The sample included 85 participants. Participants reported impairments in both, occupational functioning, with 58.7% not returned to work (RTW), and HRQoL, specifically with social activities (37%), anxiety (34%), and cognitive function (34%). Women had significantly (*p* < 0.05) worse physical symptoms (sleep disturbance and fatigue), emotional health (depression, stigma, emotional dyscontrol) scores, and sense of self-identify post-stroke. Over 70% of participants preferred in-person post-stroke care led by health care providers and felt they would have benefited from receiving information on physical health (71.4%), emotional and psychological health (56.0%), RTW (38.1%), and self-identity (26.2%) post-stroke. Women preferred cognitive behavioral therapy (*p* = 0.018) and mindfulness-based stress reduction therapy (*p* = 0.016), while men preferred pharmacotherapy (*p* = 0.02) for psychological symptoms.

**Conclusion:**

This is the first study to report impaired HRQoL, psychological and occupational functioning using PROMs, with significant gender differences and preferences for post-stroke care delivery among young adult stroke patients at >90 days after stroke. The findings highlight the importance of needs, gender, and age-specific post-stroke education and interventions.

## 1 Introduction

Over the past three decades, the global incidence of stroke has increased by 70%, making stroke a major global public health issue (Feigin et al., 2021). Traditionally, stroke has been considered a disease of older adults (Yahya et al., 2020); however, epidemiological evidence suggests an emerging trend of increasing stroke incidence among young adults (≤ 65 years) by 40% over the past decade globally (Béjot et al., 2016; Ekker et al., 2018). A potential explanation for the surge in stroke incidence is the rising prevalence of modifiable risk factors such as hypertension, poor diet, dyslipidemia, and low physical activity (O'Donnell et al., 2016).

The rise in stroke incidence among young adults has significant implications during the “active” period of their life. Specifically, young adults experience complex psychological, mental (e.g., depression, anxiety) (Waje-Andreassen et al., 2013; Amaricai and Poenaru, 2016; Ekker et al., 2018; Ignacio et al., 2022), physical, and cognitive deficit (e.g., processing speed, attention, executive function, memory- working, immediate and delayed) (Palmcrantz et al., 2012; Schaapsmeerders et al., 2013; Maaijwee et al., 2014; Sasikumar and Pikula, 2018), post-stroke, and impaired quality of life (QOL) (Singhal et al., 2013; Gurková et al., 2023). They also experience a negative impact on their identity, roles, relationships, social, and occupational wellbeing, coupled with the ability to drive, return to work, participate in recreational activities and engage in familial care activities (Martinsen et al., 2012; Leung et al., 2017; Walters et al., 2020; Gurková et al., 2023). This is further coupled with increase in high health care-associated costs. For instance, the average cost of a hospital stay for young adult stroke patients in the United States was $34,886 (US) for ischemic stroke, $94,482 (US) for intracerebral hemorrhage, and $146,307 (US) for subarachnoid hemorrhage (Ellis, 2010). Similar findings were noted in Australia, where the economic burden of younger stroke patients was estimated to be $2.0 billion (AUD) over 5 years, equating to $149,180 (AUD) per patient (Tan et al., 2022).

Historically, stroke outcomes have been measured using clinician-reported or “objective” assessments and measures [e.g., modified Rankin Scale (mRS) and Barthel Index (BI)] of ambulation, motor function, strength, and speech (Reeves et al., 2018; Smith et al., 2021) with limited interest in patient-reported outcome measures (PROMs). PROMS include measures of wellbeing and health-related quality of life (HRQoL) in addition to functional status, and symptom burden. This is further coupled with the current design and delivery of stroke care and services focused on physical function and conducting activities of daily living (Lawrence, 2010; Keating et al., 2021). Such a focus reflects older adults' needs and priorities (Lawrence, 2010) and fails to meet the unique and differing needs of young adult stroke patients (Shipley et al., 2020).

There is a dearth of research exploring post-stroke PROMs as well as rehabilitative preferences and needs in young adult stroke patients, and even less that examine gender differences in this population. This limits our ability to develop interventions/programs across the stroke care continuum in changing stroke demographics (Keating et al., 2021). PROMs offer a new and innovative frontier for stroke outcome-related assessments in both the clinical and research contexts as patients are placed at the center and afforded the opportunity to directly convey their perspectives of the post-stroke impact on health domains (e.g., health status, physical, psychological, function, emotional and mental), QOL, and outcomes (Reeves et al., 2018). Therefore, the purpose of this study was to assess: (1) young adult stroke patients' physical, psychological, and occupational functioning and HRQoL; and (2) post-stroke care preferences using PROMs, with attention to gender differences in these domains.

## 2 Methods

### 2.1 Study design

A cross-sectional cohort pilot study design of clinical and survey data from patients who attended the Stroke Prevention Clinic (SPC) at the Toronto Western Hospital (TWH), University Health Network (UHN) was used. Study findings are reported using the Strengthening the Reporting of Observational Studies in Epidemiology (STROBE) guideline for cross-sectional studies (Von Elm et al., 2008).

### 2.2 Setting

The study was conducted at the TWH, which is one of the largest stroke centers in Ontario, Canada and an academic health sciences center affiliated with the University of Toronto. A consecutive sampling method of patients attending the SPC was applied from February 2019–August 2021 with a temporary termination of participant recruitment due to the COVID-19 pandemic and a modification to the protocol. Participants who met the selection criteria were recruited by a research coordinator.

### 2.3 Study participants

The study population consisted of young adult stroke patients, who were eligible if they were, at the time of participation: (1) of working age (<65 years of age); and (2) had an ischemic or hemorrhagic stroke >90 days prior to recruitment. Patients were excluded from the study if they: (1) had advanced cognitive or functional impairment that would limit the provision of informed consent or participation (defined as having a diagnosis of dementia and/or mRS > 4); (2) had moderate to severe aphasia based on the National Institutes of Health Stroke Scale (NIHSS) item #9; (3) communicated in a language other than English; and (4) had a subarachnoid hemorrhage or stroke due to trauma. The health care providers (e.g., Nurse Practitioner or Neurologist) considered patients for inclusion based on their assessments and referred them to the research coordinator. Following this, the research coordinator approached potential participants to describe the study purpose, provided an information letter, answered any clarifying questions, and attained written, informed consent from those interested in taking part in the study.

### 2.4 Ethical considerations

The research study was approved by the UHN Research Ethics Board (REB # 17-6092). Written consent was obtained from all participants.

### 2.5 Data collection

Data were collected from the patients who consented to participate in the study and were collected from the patient's charts and through a survey (either hard copy or online, based on participant preference). The following section describes the quantitative data collection and analysis procedures and instrumentation.

#### 2.5.1 Sociodemographic and clinical characteristics

Sociodemographic and clinical characteristics were collected either through the survey or chart review from the electronic records by the research coordinator. Participants' *sociodemographic characteristics* were assessed with questions about age, sex, gender, ethnicity, level of education, living arrangement, marital status, family structure, and pre- and post-stroke income (individual and household), and insurance status. Participants' *clinical characteristics* were assessed with questions or were collected from the medical charts about pre- and post- health conditions (including physical, surgical, psychiatric, and substance use disorders), current medications, time since stroke, stroke type, etiology, severity (NIHSS) (NIH Stroke Scale), first or recurrent stroke, acute stroke care, mRs (Banks and Marotta, 2007), Montreal Cognitive Assessment (MOCA) (Hobson, 2015), and receipt of other rehabilitative, medical, and/or mental health care following stroke.

#### 2.5.2 Variables and instrumentation

The main study variables were: (1) occupational functioning; (2) physical, psychological, and social wellbeing; and (3) preferences for post-stroke care and interventions/programs.

##### 2.5.2.1 Occupational functioning

Data were obtained through questions on pre-and post-stroke occupation (manual labor, professional and clerical, semi-skilled professional, and other), pre-and post-stroke work hours, and/or post-stroke work status.

##### 2.5.2.2 Physical, psychological, and social wellbeing

The Quality of Life in Neurological Disorders (Neuro-QoL) short-form tool (Cella et al., 2012) was used. The Neuro-QoL is a multi-dimensional patient reported measurement tool that assesses the mental, physical and social health of adults and children living with neurological conditions (Cella et al., 2012). The short Neuro-QoL comprises of 13 domains with 5–9 items in each domain anchored on a 5-point Likert scale (Cella et al., 2012). The main domains are: (1) *physical* (function: upper extremity function-fine motor, lower extremity function-mobility, & symptoms: sleep disturbance, fatigue measures); (2) *psychological* (emotional health: anxiety, depression, positive affect and wellbeing, stigma, emotional and behavioral dyscontrol, cognitive health: cognition function and communication); and (3) *social* (ability to participate and be satisfied with social roles/activities) wellbeing. In accordance with the scoring methods for the Neuro-QoL short-form tool, the raw scores were converted to T scores, which are standardized to a Mean of 50 and a Standard Deviation (SD) of 10 (Cella et al., 2012). The following subdomains were scored *positively*, such that higher scores reflect better functioning: lower (mobility) and upper extremity (fine motor) function, applied cognition-executive function, applied cognition-general concerns, positive affect and wellbeing, social roles, and activities, as well as satisfaction with social roles and activities. Whereas, the subdomains of fatigue, sleep disturbance, anxiety, depression, emotional and behavioral dyscontrol, and stigma were scored *negatively*, such that higher scores reflect poorer functioning (Cella et al., 2012). The Neuro-QoL short-form tool (Cella et al., 2012) is clinically relevant and psychometrically robust (Cronbach's α = 0.85–0.97) (Cella et al., 2012) in assessing HRQoL among persons living with neurological disorders.

##### 2.5.2.3 Care preferences

Participants were asked questions about: (1) preferences with focus on post-stroke care (e.g., pharmacotherapy, cognitive behavioral therapy, mindfulness-based stress reduction); (2) areas that would be beneficial to receive advice or assistance (e.g., emotional and psychological health, romantic/sexual relationship, RTW, childcare issues); and (3) preferred method of delivery of support (e.g., in-person, telemedicine, person-led, peer-based) for post-stroke care.

### 2.6 Statistical methods

Descriptive and inferential statistics were conducted using the R software v4.1.0 (R Core Team, 2022). Descriptive statistics were used to describe the sample, specifically the sociodemographic and clinical characteristics, HRQoL coupled with psychosocial and occupational functioning. Continuous variables were described using means and SDs and categorical variables were described using counts and percentages. The proportion of participants with impaired Neuro-QoL scores in each domain were determined. Linear Model ANOVA's for normally distributed continuous data and Pearson chi-square tests for categorical data were used for the inferential statistics. Across each Neuro-QoL domain, univariable associations between age (continuous and categorical <50 or = > 50), gender (women/men), time post-stroke (continuous), mRS (0, 1, 2, 3) (Webster et al., 2011), RTW (yes, no), and post-stroke care (yes, no) were examined between participants with and without impaired HRQoL. Return to work was treated as a dichotomous variable, with any paid work being assigned as a positive RTW (Dreyer et al., 2016). The significance level for all analyses was set at *p* < 0.05.

Multivariable logistic regression analyses were also conducted to investigate the relationship between variables that were significantly associated with impaired HRQoL in the univariable analysis. By developing these models, the characteristics that had the greatest impact on HRQoL and functioning following stroke and, therefore, predicting those at highest risk of poor outcomes in these domains were identified. Gender was included as predictor variable in the multivariable models with a focus on the evaluation of the association between sociodemographic/clinical characteristics and HRQoL and RTW. Multicollinearity was assessed in each model using variance inflation factor and model fit was evaluated using McFadden's Pseudo-R squared. A McFadden's *R*^2^ of 0.2–0.4 suggests excellent model fits (McFadden, 1977).

## 3 Results

### 3.1 Participants

**Figure 1:**
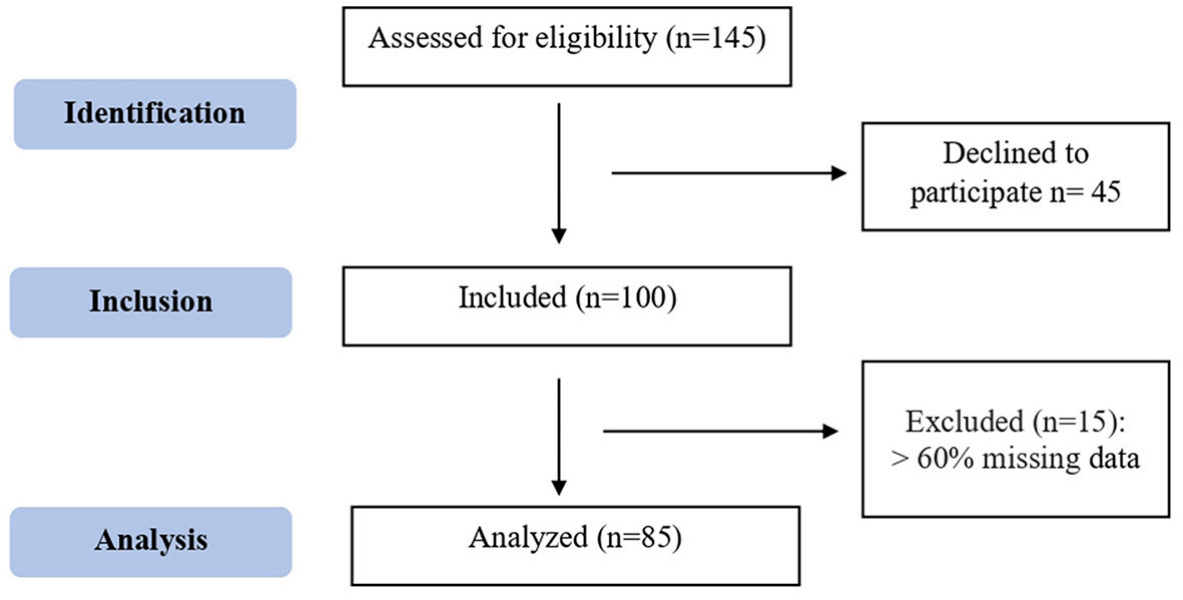
STROBE flow chart.

A total of 145 patients were assessed for eligibility and of those, 45 declined to take part in the study. Of the 100 participants included, 15 were excluded for having >60% missing data, resulting in a final sample of 85 young adult stroke patients (Figure 1). All participants were cis gender meaning they identified with their biological sex with the exception for one participant who did not answer the gender question; resulting in 84 participants being included in the gender analysis. Over half of the participants were men (58.3%) with a mean age of 48.3 years (SD: 11.2, range 21–65). Men were significantly older than women, with a mean age difference of 7.7 years (*p* = 0.001). Participants reported being white (59.5%), married (54.7%), and having advanced degrees (55.4%). Additionally, the participants' family structure varied based on gender with women more likely to live with their mothers (*p* = 0.002) and men with their fathers (*p* = 0.002) (Table 1).

**Table 1:** Demographic and clinical characteristics.

	**Women (*n* = 35)**	**Men (*n* = 49)**	**Total (*N* = 84)**	***p*-value**
**Age**				**0.001 (1)**
Mean (SD)	43.3 (10.6)	51.5 (10.3)	48.3 (11.2)	
Range	21.0–63.0	26.0–65.0	21.0–65.0	
**Ethnicity**				
White	21 (60.0%)	29 (59.2%)	50 (59.5%)	0.940 (2)
African	0 (0.0%)	6 (12.2%)	6 (7.1%)	**0.032 (2)**
Asian	4 (11.4%)	3 (6.1%)	7(8.3%)	0.386 (2)
Hispanic	3 (8.6%)	0 (0.0%)	3 (3.6%)	**0.037 (2)**
Other	5 (14.3%)	5 (10.2%)	10 (11.9%)	0.569 (2)
**Insurance Status**				0.823 (2)
Missing	8	5	13	
Advanced	13 (48.1%)	20 (45.5%)	33 (46.4%)	
Basic	6 (22.2%)	8 (18.2%)	14 (19.7%)	
None	9 (29.6%)	16 (36.4%)	25 (33.8%)	
**Marital Status**				0.729 (2)
Missing	4	5	9	
Living with partner	3 (9.7%)	5 (11.4%)	8 (10.7%)	
Married	18 (58.1%)	23 (52.3%)	41 (54.7%)	
Never married	8 (25.8%)	10 (22.7%)	18 (24.0%)	
Separated/ divorced	2 (6.5%)	6 (13.6%)	8 (10.7%)	
**Living arrangement**				0.674 (2)
Missing	3	5	8	
Assisted living facility	0 (0.0%)	1 (2.3%)	1 (1.3%)	
Home	29 (90.6%)	41 (93.2%)	70 (92.1%)	
Nursing facility	1 (3.1%)	1 (2.3%)	2 (2.6%)	
**Family structure**				
Mother	8 (22.2%)	1 (2.0%)	9 (10.7%)	**0.002 (2)**
Father	2 (5.7%)	17 (34.7%)	19 (22.6%)	**0.002 (2)**
Siblings	1 (2.9%)	1 (2.0%)	2 (2.4%)	0.809 (2)
Partner	5 (14.3%)	6 (12.2%)	11 (13.1%)	0.785 (2)
Children	15 (42.9%)	14 (28.6%)	29 (34.5%)	0.175 (2)
Grandchildren	0 (0.0%)	1 (2.0%)	1 (1.2%)	0.395 (2)
Other	2 (5.7%)	2 (4.1%)	4 (4.8%)	0.729 (2)
**Highest level of education**				0.321 (2)
Missing	3	7	10	
Advanced degree	19 (59.4%)	22 (52.4%)	41 (55.4%)	
College degree	7 (21.9%)	8 (19.0%)	15 (20.3%)	
High school or equivalent	2 (6.2%)	6 (14.3%)	8 (10.8%)	
Some college	3 (9.4%)	1 (2.4%)	4 (5.4%)	
Some high school or less	1 (3.1%)	5 (11.9%)	6 (8.1%)	
**Time since stroke (no. days)**				0.794 (1)
Missing	2	5	7	
Mean (SD)	348.1 (478.4)	324.2 (321.2)	334.4 (393.6)	
**Stroke type**				0.696 (2)
Missing	4	7	11	
Hemorrhagic	9 (29.0%)	14 (33.3%)	23 (31.5%)	
Ischemic	22 (71.0%)	28 (66.7%)	50 (68.5%)	
**Recurrent stroke**				0.289 (2)
Missing	6	23	29	
No	24 (82.8%)	24 (92.3%)	48 (87.3%)	
Yes	5 (17.2%)	2 (7.7%)	7 (12.7%)	
**Rehabilitation program- post stroke**				0.285(2)
Missing	21	36	57	
Education	0 (0.0%)	1 (7.7%)	1 (3.7%)	
Home	1 (7.1%)	4 (30.8%)	5 (18.5%)	
None	3 (21.4%)	1 (7.7%)	4 (14.8%)	
Rehabilitation	9 (64.3%)	7 (53.8%)	16 (59.3%)	
Speech rehabilitation	1 (7.1%)	0 (0.0%)	1 (3.7%)	
**Rehabilitation type**				0.222 (2)
Missing	7	19	26	
Home	14 (50.0%)	20 (66.7%)	34 (58.6%)	
Inpatient	5 (17.9%)	6 (20.0%)	11 (19.0%)	
Outpatient	9 (32.1%)	4 (13.3%)	13 (22.4%)	
**MoCA**				0.495 (1)
Missing	17	22	39	
Mean (SD)	28.4 (2.0)	28.0 (2.5)	28.2 (2.3)	
Range	23–30	20–30	20–30	
**Poststroke modified Rankin Score (mRS)**				
Mean (SD)	0.7(0.7)	0.7(0.6)	0.7(0.6)	0.821(1)
**Modified Rankin Score**				0.066 (2)
Missing	12	22	34	
0	5 (21.7%)	12 (44.4%)	17 (34.0%)	
1	11 (47.8%)	11 (40.7%)	22 (44.0%)	
2	6 (26.1%)	1 (3.7%)	7 (14.0%)	
3	1(4.3%)	3(11.1%)	4(8.0%)	

1. Linear model ANOVA. 2. Pearson's chi-squared test.

For the participants' *clinical characteristics*, 68.5% had an ischemic stroke, and significantly more men had a hemorrhagic stroke than women (*p* = 0.047). The mean time of post-stroke was 334 days (equivalent to 11 months) with participants having a mild stroke (NIHSS 3) and good cognitive outcomes (MOCA 28). Many participants (58.6%) reported receiving rehabilitation at home post-stroke, and the post-stroke mRS score varied with 44.0% scoring 1 (no significant disability despite symptoms) (Table 1).

### 3.2 Occupational functioning

The post-stroke status of participants was mixed with 58.7% not RTW. A much lower percentage of participants (13.9%) were working 30–40 h/week post-stroke compared to pre-stroke (44.6%). Further, a higher percentage of participants (25.7%) were unemployed post-stroke compared to pre-stroke (5.2%). There was also a change in occupation before and after stroke. Specifically, 53.2% participants reported having professional and clerical occupations pre-stroke and only 34.3% held this occupation post-stroke. Moreover, many participants were the primary earners in their household (54.5%) with men more likely to be primary earners compared to women (*p* = 0.021). While there were 40% missing data for individual and 60% for household income post-stroke, which is a common pattern in survey data on personal and household income (Yan et al., 2010; Daniels, 2022), 43.6% of the individual and 55.2% of the household incomes were >$100,000; with men having a higher household income pre-stroke than women (*p* = 0.069) (Table 2).

**Table 2:** Occupational characteristics.

	**Women (*n* = 35)**	**Men (*n* = 49)**	**Total (*N* = 84)**	***p*-value**
**Post-stroke work status**				0.091(1)
Missing	5	5	10	
Disability/leave	11 (36.7%)	19 (43.2%)	30 (40.5%)	
Full-time employed	11 (36.7%)	12 (27.3%)	23 (31.1%)	
Full-time student	1 (3.3%)	1 (2.3%)	2 (2.7%)	
Homemaker	2 (6.7%)	0 (0.0%)	2 (2.7%)	
Part-time employed	3 (10.0%)	6 (13.6%)	9 (12.2%)	
Retired	0 (0.0%)	6 (13.6%)	6 (8.1%)	
Unemployed	2 (6.7%)	0 (0.0%)	2 (2.7%)	
**Occupation before stroke**				0.130
Missing	3	5	8	
Manual labor	1 (3.0%)	8 (18.2%)	9 (11.7%)	
Professional & clerical	18 (54.5%)	23 (52.3%)	41 (53.2%)	
Retired	0 (0.0%)	3 (6.8%)	3 (3.9%)	
Semi-skilled professional	10 (30.3%)	7 (15.9%)	17 (22.1%)	
Student	2 (6.1%)	1 (2.3%)	3 (3.9%)	
Unemployed	2 (6.1%)	2 (4.5%)	4 (5.2%)	
**Occupation after stroke**				0.274
Missing	6	9	15	
Manual labor	1 (3.3%)	2 (5.0%)	3 (4.3%)	
On leave/disability	2 (6.7%)	5 (12.5%)	7 (10.0%)	
Professional & clerical	11 (36.7%)	13 (32.5%)	24 (34.3%)	
Retired	0 (0.0%)	5 (12.5%)	5 (7.1%)	
Semi-skilled professional	4 (13.3%)	4 (10.0%)	8 (11.4%)	
Student	4 (13.3%)	1 (2.5%)	5 (7.1%)	
Unemployed	8 (26.7%)	10 (25.0%)	18 (25.7%)	
**Return to work**				0.298 (2)
Missing	5	5	10	
No	16 (51.6%)	28 (63.6%)	44 (58.7%)	
Yes	15 (48.4%)	16 (36.4%)	31 (41.3%)	
**Primary earner in house**				**0.021 (1)**
Missing	2	5	7	
No	20 (60.6%)	15 (34.1%)	35 (45.5%)	
Yes	13 (39.4%)	29 (65.9%)	43 (54.5%)	
**Work hours before stroke**				0.619 (1)
Missing	9	10	19	
>40 per week	11 (42.3%)	18 (46.2%)	29 (44.6%)	
20–30 per week	4 (15.4%)	3 (7.7%)	7 (10.8%)	
30–40 per week	11 (42.3%)	18 (46.2%)	29(44.6%)	
**Work hours after stroke**				0.765 (1)
Missing	5	7	12	
<20 per week	18 (60.0%)	29 (69.0%)	47 (65.3%)	
>40 per week	4 (13.3%)	4 (9.5%)	8 (11.1%)	
20–30 per week	4 (13.3%)	3 (7.1%)	7 (9.7%)	
30–40 per week	4 (13.3%)	6 (14.3%)	10 (13.9%)	
**Individual income before stroke**				0.570 (1)
Missing	15	14	29	
$20–49,000	6(30.3%)	7 (20.0%)	13 (23.6%)	
$50–99,000	7 (35.0%)	11 (31.4%)	18 (32.7%)	
>$100,000	7 (35.0%)	17 (48.6%)	24 (43.6%)	
**Household income before stroke**				0.069 (1)
Missing	10	16	26	
$20–49,000	4 (16.0%)	1 (3.0%)	5 (8.6%)	
$50–99,000	11 (44.0%)	10 (30.3%)	21 (36.2%)	
>$100,000	10 (40.0%)	22 (66.7%)	32 (55.2%)	
**Individual income after stroke**				0.449 (1)
Missing	24	28	52	
$20–49,000	4 (36.4%)	6 (28.6%)	10 (31.2%)	
$50–99,000	1 (9.1%)	6 (28.6%)	7 (21.9%)	
>$100,000	6 (54.5%)	9 (42.9%)	15 (46.9%)	
**Household income after stroke**				0.091 (1)
Missing	13	22	35	
$20–49,000	8 (36.4%)	3 (11.1%)	11 (22.4%)	
$50–99,000	5 (22.7%)	11 (40.7%)	16 (32.7%)	
>$100,000	9 (40.9%)	13 (48.1%)	22 (44.9%)	

1, 2. Pearson's Chi-square test.

### 3.3 Physical, psychological, and social wellbeing (NeuroQoL)

Overall, NeuroQoL varied among the participants and across the domains, with some having undesirable self-reported health (as illustrated in Table 4). The highest impairments across the NeuroQoL (measured as a *T*-score of >55 or <45) were in the social and psychological domains; notably in the ability to participate in social roles and activities (37%), satisfaction with social roles (30%) as well as in anxiety (34%) and subjective cognitive function (34%) scores (Table 3). Gender differences within the NeuroQoL were also observed. Specifically, women reported significantly worse depression (*p* = 0.035), emotional dyscontrol (*p* = 0.015), fatigue (*p* = 0.003), stigma (*p* = 0.022), and sleep (*p* = 0.040) scores (*T*-score > 55) than men. Additionally, women had significantly worsened ability to participate in social roles (*p* = 0.020), satisfaction with social roles (*p* = 0.047), and subjective cognitive function (*p* = 0.034) scores (*T*-score <45) compared to men (Table 4).

**Table 3:** Proportion of patients with impaired QOL (T-score of >55 or <45).

**Neuro QOL domains**	**Number of participants**	**Number impaired**	**Proportion impaired (%)**
Anxiety	85	29	34
Depression	85	8	9
Ability to participate	84	31	37
Emotional	85	17	20
Fatigue	85	19	22
Lower mobility	85	11	13
Upper mobility	85	22	26
Stigma	85	14	17
Positive wellbeing	85	5	6
Satisfaction	84	25	30
Cognitive function	85	29	34
Communication	53	0	0
Sleep disturbance	53	12	23

**Table 4:** Neuro-QoL T-scores.

	**Women (*n* = 35)**	**Men (*n* = 49)**	**Total (*N* = 84)**	***p*-value**
**Anxiety**				
Mean (SD)	51.0 (8.6)	47.1 (11.4)	48.8 (10.4)	0.093 (1)
**Depression**				
Mean (SD)	46.0 (8.7)	42.4 (6.8)	43.9 (7.8)	**0.035 (1)**
**Ability to participate**				
Mean (SD)	46.1 (9.9)	50.9 (8.9)	48.9 (9.6)	**0.020 (1)**
**Emotional dyscontrol**				
Mean (SD)	46.9 (10.3)	41.2 (10.4)	43.6 (10.7)	**0.015 (1)**
**Fatigue**				
Mean (SD)	50.0 (11.6)	42.5 (10.8)	45.6 (11.7)	**0.003 (1)**
**Lower mobility**				
Mean (SD)	51.7 (7.6)	53.5 (6.8)	52.8 (7.2)	0.278 (1)
**Upper mobility**				
Mean (SD)	49.4 (7.5)	49.5 (8.5)	49.5 (8.1)	0.971 (1)
**Stigma**				
Mean (SD)	49.4 (7.5)	45.7 (6.7)	47.2 (7.3)	**0.022 (1)**
**Positive wellbeing**				
Mean (SD)	53.9 (9.0)	56.1 (8.3)	55.2 (8.6)	0.247 (1)
**Satisfaction**				
Mean (SD)	47.8 (7.0)	50.9 (7.0)	49.6 (7.2)	**0.047 (1)**
**Cognitive function**				
Mean (SD)	48.6 (10.2)	53.4 (10.0)	51.4 (10.3)	**0.034 (1)**
**Communication**				
Missing	13	19	32	
Mean (SD)	93.6 (11.6)	92.8 (12.7)	93.2 (12.1)	0.816 (1)
**Sleep**				
Missing	13	19	32	
Mean (SD)	46.4 (11.9)	39.6 (11.1)	42.5 (11.8)	**0.040 (1)**

1. Linear Model ANOVA. Bold values indicate statistically significant.

In the multiple regression analysis, when controlling for confounders (gender, RTW), the longer the time since stroke (11 months), the more impaired anxiety *T*-scores (*p* = 0.03). Second, age was associated with impaired Upper Extremity Function *T*-scores, specifically increase in age (OR, 1.08; 95% CI 1.02–1.15). Finally, women (OR, 6.57; 95% 1.54 27.81) and older participants (aged 50–65) (OR, 5.82; 95% 1.32 25.71) had greater odds of impaired Stigma *T*-scores (Table 5).

**Table 5:** Multivariable regression analysis.

**Variable**	**Number of impaired QOL/total number of cases**	**OR (95% CI)**	***p*-value**
**Anxiety** ***T*****-Score** > **55**			
**Gender**			
Male	13/67	Reference	–
Female	12/67	1.36 (0.47–3.97)	0.57
**Time since stroke**			
Continuous	–	1.00 (1.00–1.00)	**0.03**
**Return to work**			
No	15/67	Reference	–
Yes	11/67	0.92 (0.32–2.69)	0.88
**Upper extremity function- fine motor** ***T*****-Score of**<**45**			
**Gender**			
Men	12/74	Reference	–
Women	10/74	3.05 (0.83–11.2)	0.12
**Age**			
Continuous	–	1.08 (1.02–1.15)	**0.01**
**Return to work**			
No	14/74	Reference	–
Yes	6/74	0.34 (0.09–1.17)	0.07
**Stigma** ***T*****-Score** > **55**			
**Gender**			
Men	5/77	Reference	–
Women	9/77	6.57 (1.54–27.81)	**0.02**
**Time since stroke**			
Continuous	–	1.00 (0.99–1.00)	0.40
**Age**			
<50	4/77	Reference	
50–65	10/77	5.82 (1.32–25.71)	**0.03**

Bold values indicate statistically significant.

### 3.4 Care preferences

Participant's care preferences for areas of focus and method of delivery of post-stroke care varied (Appendix 1). Specific to *areas of focus*, over 70% of participants reported the need for additional support for their physical health, 56% for their emotional and psychological health support, 38% for the ability to RTW, and 26.2% on post-stroke self-identity. Notably, women (37.1%) preferred additional support with focus on self-identity post-stroke compared to men (18.4%) (*p* = 0.05).

Women also significantly preferred cognitive behavioral therapy (*p* = 0.018) and mindfulness-based stress reduction (0.016) as areas to focus on during post-stroke care compared to men. Whereas, men significantly preferred (p = 0.02) pharmacotherapy as an area of focus in post-stroke care compared to women. Furthermore, women (37.1%) preferred additional support with self-identity post-stroke compared to men (18.4%) (*p* = 0.05). Specific to *method of delivery for post-stroke care*, over 70% of participants indicated in-person as their preference as well as person-led (66.7%) and professional-led (54.8%) support. Gender differences with preferred method of delivery for post-stroke care was also significant, such that more women preferred on-demand (*p* = 0.015) and in-person support (*p* = 0.054) compared to men (Supplementary Table 1).

## 4 Discussion

**Figure 2:**
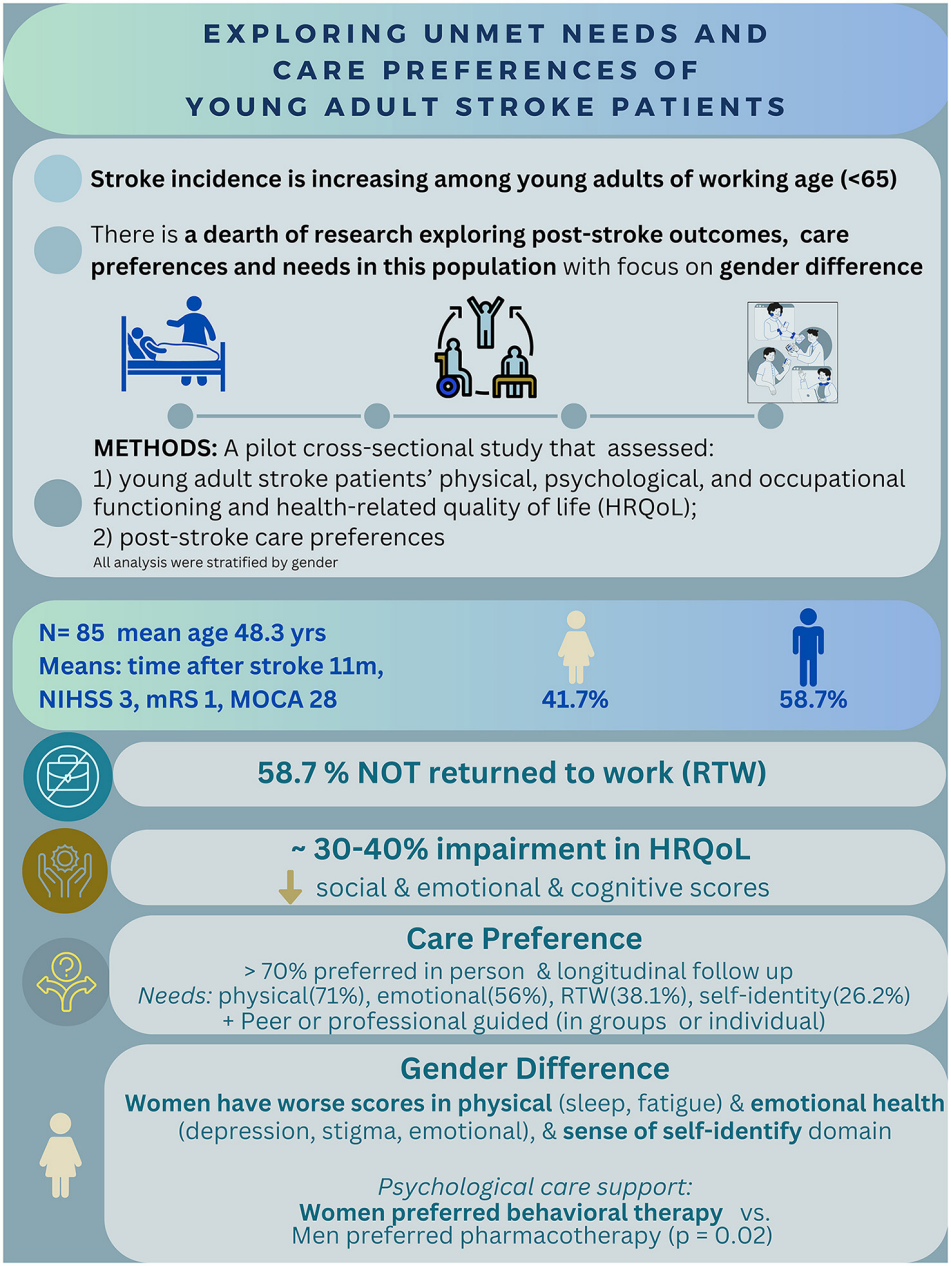
Summary of findings.

The provision of person-centered and high-quality stroke care to all patients is challenging, but particularly to young adult stroke patients. Young adult stroke patients experience a profound diversion from their life trajectory of building their career and caring for their family, to compromised occupational and psychosocial functioning as well as HRQoL. The findings from this study highlight the multifaceted challenges and impairments that young adult stroke patients experience post-stroke (Figure 2).

### 4.1 Occupational functioning and HRQoL domains

In this study, *occupational functioning* changed among young adult stroke patients with 58.7% not RTW, 34.3% (from 53.2% pre-stroke) having returned to their professional and clerical positions, and only 4.3% returned to their manual labor occupation post-stroke (from 11.7% pre-stroke). Of note, the rate of RTW among participants in this study was higher than previously observed in the literature. For instance, among young adult stroke adult participants in the Helsinki Young Stroke registry-based follow-up study (Aarnio et al., 2018), 37.6% were not working at 1-year, 42.0% at 2-years, and 46.9% at 5-years, post-stroke. The associations of lower rates of RTW in the Helsinki Young Stroke study included men, blue-collar workers, and having a higher degree of functional disability (moderate to severe aphasia, mild, moderate or severe limb paresis, moderate to severe visual field deficit) (Aarnio et al., 2018). This is contrary to our study where participants had milder stroke, good functional and cognitive outcomes, and the majority were discharged home, did not attend any inpatient or outpatient rehabilitation, and were of higher socio-economic status. Similar findings were also echoed in a systematic review that determined the frequency and predicting factors of RTW for young adult stroke patients (18–65 years) (Edwards et al., 2018). In the systematic review, the median frequency of RTW increased with time- from 41% zero to 6 months, 53% 1-year, 56% 1.5-years, and 66% 2–4-years post-stroke (Edwards et al., 2018). Furthermore, predicting factors of RTW included men, greater independence in activities of daily living, better cognitive ability, fewer neurological deficits, and employment in a professional/office setting (Edwards et al., 2018).

Impairments across the *HRQoL domains* were also observed in this study and mostly related to the social and mental domains despite good MOCA scores. This is important to note because although this cohort had mild strokes and otherwise good functional outcomes, they still experienced HRQoL-related impairments which extended beyond the physical and functional status (motor, language or cognitive), and to more complex psychological outcomes. While the cross-sectional pilot design limits our understanding of contributing factors for the observed impairments, there are several potential explanations to the occupational and HRQoL-related impairments. First, appreciating that at the time of the stroke event, adults under 65 years of age are often building their careers and working toward their respective professional goals. The premature and unanticipated exit from the labor markets and increased reliance on government subsidy post-stroke can be challenging for young adults who may have dependents (e.g., children, aging parents) and other financial demands (e.g., mortgage). This in turn, may impair their ability to meet physical, psychological and social needs, and potentially affect their overall health, quality of life, wellbeing, self-esteem, life satisfaction, sense of achievement and facilitation of individual identity, autonomy, security, and social status (Baldwin and Brusco, 2011; Morris, 2011; Edwards et al., 2018; La Torre et al., 2022). Second, the higher levels of anxiety and poor subjective cognitive function may be attributed to the uncertainty of the long-term outcomes, potential complications, and fear of stroke re-occurrence (Opoku et al., 2020). Third, while the data were not collected in this study, there is growing research on biological mechanisms such as neuroinflammation and infection potentially contributing to cognitive impairment post-stroke and overall psychological outcomes (Kliper et al., 2013; Milosevich et al., 2023). Fourth, having to develop a new and/or adjusted identity post-stroke. Finally, the reconciliation of having an “*older person's disease*” and receiving care that is geared toward older persons (Dale Stone, 2005). Such reconciliation is further amplified with current post-stroke rehabilitation programs primarily focusing on regaining physical function and activities of daily living (Lawrence, 2010; Keating et al., 2021) and not the psychological outcomes and the RTW process (Shipley et al., 2020). In addition, young adult stroke patients with mild post-stroke symptoms may not require post-stroke services, and are often discharged home with minimal or no referral to post-stroke interventions resulting in less attention to support their unique and long-term post-stroke needs (Wolfenden and Grace, 2015; Maratos et al., 2016).

In this study, we also observed gender differences across most of the HRQoL domains; specifically, in social (ability to participate in social activities) and physical health (fatigue and sleep), but more so in mental health (emotional health: anxiety, fatigue, stigma, cognitive health: subjective cognitive function), with women experiencing worse symptoms compared to men. It has been reported that women experience a disproportionate burden of stroke-related disability, psychosocial and mental (e.g., depression) challenges, and mortality (Shi et al., 2017; Rexrode et al., 2022) compared to men. In a recent review on PROMs post-stroke, sex disparities with females experiencing more activity limitations and post-stroke depression, and worse HRQoL compared to males (Gall et al., 2018) were found, but it is important to note that most of the studies in this review included an older adult patient population with more severe strokes.

### 4.2 Care preferences

In this study, many participants highlighted numerous care preferences and methods of delivery for post-stroke interventions that centered around non-pharmacological, psychological, and emotion-based to aid with their post-stroke care and recovery. There were also gender differences in the preferred care interventions post-stroke in our study. The care preferences in stroke recovery among the young adult population has seldomly been reported in the literature. Of the limited literature that exists, three themes emerged in a qualitative study that explored the unmet needs of young adults (*n* = 19, age: 19–54 years, 6 months to 24 years from stroke event) with varying stroke severity and post-stroke functional ability in inpatient and outpatient stroke care in Australia (Shipley et al., 2020). The themes were: (1) limited focus on psychological and cognitive management; (2) limited provision of information and structured peer support; and (3) receiving needs, gender and age-specific patient care (Shipley et al., 2020). While there has been a focus in recent years on the development of app-based and online mental health supports (Neary and Schueller, 2018), the participants in our study preferred an in-person method of post-stroke care delivery and approximately half preferred care being delivered by a health care provider (HCP) compared to a peer or self-led. Such findings are consistent with an international cross-sectional study of 171 young stroke patients (age 18–55 years; mRS ranging from 1 to 3; and time since stroke: median 35.5 months), where expressed needs centered around having face-to-face contact with HCPs, meeting outside of the “traditional” health care setting as well as the provision of information through a list of tips, and peer support (Keating et al., 2021).

The ultimate purpose of exploring the needs and preferences of this unique patient population is to improve post-stroke recovery, delivery of stroke care and interventions/programs, in addition to models of stroke care that are currently geared toward older adult stroke patients. Our study comprehensively examined the unmet needs of young adult stroke patients across all relevant long-term post-stroke outcomes such as HRQoL, psychological and occupational functioning, as well as care preferences. The findings have several implications to future design of post-stroke recovery and clinical care practice that encompasses age and gender more carefully.

First, enhancing HCPs' knowledge and understanding, directly from the patients' perspective of their health status, gender differences, and novel ideas for targeting post-stroke care interventions in a manner using PROMs (Reeves et al., 2018). Second, the importance of integrating an intersectionality framework in the development of needs, gender and age-specific post-stroke education and interventions appreciating how social identity may influence access, navigation, affordance, and biases; all of which may contribute to stroke outcomes (Berkhout et al., 2024). For example, their personal (e.g., parenting, driving, self-identity, social participation) and professional (e.g., RTW, career growth and development) life stages. Third, the development of non-pharmacological and peer support interventions for the non-physical aspects of stroke (e.g., self-identity, emotions, role, social participation) based on key goals of this patient population (e.g., RTW, remaining active in social and family lives, maintaining a career) (Sasikumar and Pikula, 2018). Interestingly, non-pharmacological and age-specific education and interventions are common in oncology with Adolescent and Young adult (AYA) programs (Haines et al., 2023) and a similar structure in stroke care would be beneficial particularly because stroke interventions/programs have traditionally focused on physical and cognitive rehabilitation as the essential aspects of stroke recovery. However, based on the study findings and supported by the literature, young adult stroke patients continue to have issues with other aspects of their recovery and functioning that are, perhaps, not targeted by traditional post-stroke rehabilitation interventions/programs. Finally, the importance of employing a co-design, participatory approach in the development of post-stroke-related education and interventions/programs (Batalden et al., 2016; Auger et al., 2022) to ensure they are person-centered, effective, sustainable, and appropriate in overcoming the significant challenges and barriers experienced by this unique patient population post-stroke.

The study has several limitations. First, the inclusion criteria were limited to persons without or with onlymild aphasia, and who were able to communicate in English. This limits our ability to generalize the study findings to other young adult stroke patient populations who may have additional and/or more targeted needs. Second, 15 participants were excluded from the data analysis for having >60% missing data and their responses may have differed from those comprising the final study sample. Third, this pilot study was conducted in a single center in Toronto, Ontario, Canada, which may limit the generalizability of the findings although stroke and post-stroke service in Canada is universally designed around the stroke best practice guidelines. Fourth, the definitions of several variables included in this study, such as young adult stroke patient and RTW status, are inconsistently defined in the literature, yet we wanted to capture the post-stroke needs across the age of the “working population”. Although we based our operationalization of these variables on past literature, it is important to note that the variability that exists across studies may make comparison with the literature challenging. Fifth, there was ≤ 40% of non-responses for post-stroke individual and household income-related questions, however, it is important to note that such finding is a common trend in the survey data due to the sensitivity of the questions particularly when related to loss of income and the perceived missing data may represent participant's unwillingness and lack of comfort with answering these sensitive questions (Yan et al., 2010; Daniels, 2022). Finally, Ontario has a universal health care system, and many aspects of care are paid for, which may be limit generalizability of the study findings to other jurisdictions where engagement in post-stroke care is not provided or may vary in duration. However, longer term care (beyond 6 months post-stroke) and non-physician psychological care are generally not covered by the government, as such, engagement in this care may be impacted by their ability to pay and/or private insurance coverage. As participants faced these system limitations during the period of their involvement in the study, this may have impacted some of their responses about preferences.

## 5 Conclusion

Stroke rates among young adults have increased dramatically in the past decade. Appreciating that existing literature, guidelines, and model of stroke care tend to focus on older adults who have differing needs and priorities to young adults. The study sought to explore young adults' HRQoL, psychological, and occupational functioning using PROMs as well as preferences on post-stroke support and interventions/programs. There were noted impairments in psychological and occupational functioning as well as gender differences with Neuro-QOL, preferences for post-stroke interventions/program. The study findings have implications on the importance of enhancing HCPs' knowledge and understanding the unmet needs of this unique patient population, developing needs, age and gender-specific education and post-stroke-related interventions/programs (that focus more on RTW and the psychosocial aspect of stroke). The incorporation of PROMs is critical in health care services to ensure person-centered, high-quality care and post-stroke interventions/programs are informed by patients, as the persons with the lived experience, with the ultimate goals of improving health and outcomes in addition to reconceptualizing the current model of stroke care and policy.

## Data availability statement

The raw data supporting the conclusions of this article will be made available by the authors, without undue reservation.

## Ethics statement

The studies involving humans were approved by UHN Research Ethics Board (REB # 17-6092). The studies were conducted in accordance with the local legislation and institutional requirements. The participants provided their written informed consent to participate in this study.

## Author contributions

SI: Writing – original draft, Writing – review & editing, Data curation, Formal analysis. TF: Data curation, Formal analysis, Writing – original draft, Writing – review & editing. KS: Conceptualization, Supervision, Writing – original draft, Writing – review & editing. KK: Writing – original draft, Writing – review & editing. AS: Writing – original draft, Writing – review & editing. SH: Writing – original draft, Writing – review & editing. CK: Writing – original draft, Writing – review & editing. SN: Data curation, Writing – original draft, Writing – review & editing. SB: Writing – original draft, Writing – review & editing. JC: Writing – original draft, Writing – review & editing. VR: Formal analysis, Methodology, Writing – original draft, Writing – review & editing. AP: Conceptualization, Data curation, Formal analysis, Funding acquisition, Investigation, Resources, Supervision, Writing – original draft, Writing – review & editing.
