# Supplementary material for: Exploring unmet needs and preferences of young adult stroke patients for post-stroke care through PROMs and gender differences
Source: Front Stroke. 2024 May 9;3:1386300. doi: 10.3389/fstro.2024.1386300 (PMC12802803; doi:10.3389/fstro.2024.1386300)
Supplement: Supplementary file 1 [file Table_1.DOCX]

Supplementary Table 1. Patient preferences of post stroke care

|  | **Women**  **(n=35)** | **Male**  **(n=49)** | **Total**  **(N=84)** | **p value** |
| --- | --- | --- | --- | --- |
| **Preferred Area of Focus in Follow-Up Care** |  |  |  |  |
| **Pharmacotherapy** |  |  |  |  |
| Missing | 3 | 7 | 10 | **0.020 (2)** |
| Mean (SD) | 53.5 (43.6) | 75.8 (36.6) | 66.1 (41.1) |  |
| **Problem Solving Therapy** |  |  |  |  |
| Missing | 3 | 9 | 12 | 0.397 (2) |
| Mean (SD) | 75.3 (37.8) | 67.5 (39.3) | 71.0 (38.6) |  |
| **Cognitive Behavioural Therapy** |  |  |  |  |
| Missing | 4 | 10 | 14 | **0.018 (2)** |
| Mean (SD) | 89.6 (23.1) | 70.5 (38.7) | 79.0 (33.9) |  |
| **Interpersonal Therapy** |  |  |  |  |
| Missing | 5 | 10 | 15 | 0.118 (2) |
| Mean (SD) | 72.3 (32.9) | 58.0 (39.9) | 64.2 (37.5) |  |
| **Solution Focused Therapy** |  |  |  |  |
| Missing | 3 | 9 | 12 | 0.229 (2) |
| Mean (SD) | 87.2 (25.1) | 78.6 (33.6) | 82.4 (30.2) |  |
| **Mindfulness Based Stress Reduction** |  |  |  |  |
| Missing | 3 | 9 | 12 | **0.016 (2)** |
| Mean (SD) | 83.5 (31.6) | 61.2 (42.6) | 71.1 (39.5) |  |
| **Yoga** |  |  |  |  |
| Missing | 3 | 9 | 12 | 0.465 (2) |
| Mean (SD) | 59.3 (45.6) | 51.9 (39.6) | 55.2 (42.2) |  |
| **Music Therapy** |  |  |  |  |
| Missing | 4 | 9 | 13 | 0.665 (2) |
| Mean (SD) | 65.8 (41.4) | 61.5 (41.1) | 63.4 (41.0) |  |
| **What specific area(s) do you think you would have benefitted receiving assistance/advice in?** |  |  |  |  |
| Physical health | 25 (71.4%) | 35 (71.4%) | 60 (71.4%) | 1.000 (1) |
| Emotional and psychological health | 21 (60.0%) | 26 (53.1%) | 47 (56.0%) | 0.528 (1) |
| Caregiving/family relationship | 7 (20.0%) | 7 (14.3%) | 14 (16.7%) | 0.488 (1) |
| Romantic/sexual relationship | 7 (20.0%) | 6 (12.2%) | 13 (15.5%) | 0.333 (1) |
| Sense of self and identity after stroke | 13 (37.1%) | 9 (18.4%) | 22 (26.2%) | **0.054 (1)** |
| Return to work | 15 (42.9%) | 17 (34.7%) | 32 (38.1%) | 0.448 (1) |
| Childcare issues | 3 (8.6%) | 4 (8.2%) | 7 (8.3%) | 0.947 (1) |
| None of the above | 3 (8.6%) | 4 (8.2%) | 7 (8.3%) | 0.947 (1) |
| **What would be your preferred method of delivery of this type of support?** |  |  |  |  |
| In-person | 24 (68.6%) | 35 (71.4%) | 59 (70.2%) | 0.778 (1) |
| Via phone | 8 (22.9%) | 14 (28.6%) | 22 (26.2%) | 0.557 (1) |
| Telemedicine | 11 (31.4%) | 14 (28.6%) | 25 (29.8%) | 0.778 (1) |
| Online modalities | 5 (14.3%) | 6 (12.2%) | 11 (13.1%) | 0.785 (1) |
| Person-led | 24 (68.6%) | 32 (65.3%) | 56 (66.7%) | 0.754 (1) |
| Referral out | 4 (11.4%) | 4 (8.2%) | 8 (9.5%) | 0.615 (1) |
| Scheduled | 2 (5.7%) | 3 (6.1%) | 5 (6.0%) | 0.938 (1) |
| On-demand | 4 (11.4%) | 0 (0.0%) | 4 (4.8%) | **0.015 (1)** |
| In-person support (group) | 0 (0.0%) | 4 (8.2%) | 4 (4.8%) | 0.083 (1) |
| In-person support (individual) | 5 (14.3%) | 20 (40.8%) | 25 (29.8%) | 0.009 (1) |
| In-person support (combination) | 18 (51.4%) | 15 (30.6%) | 33 (39.3%) | **0.054 (1)** |
| Person-led support (peer-led) | 9 (25.7%) | 10 (20.4%) | 19 (22.6%) | 0.567 (1) |
| Person-led support (professional-led) | 19 (54.3%) | 27 (55.1%) | 46 (54.8%) | 0.941 (1) |
| Person-led support (self-led) | 3 (8.6%) | 2 (4.1%) | 5 (6.0%) | 0.391 (1) |

1. Linear Model ANOVA
2. Pearson’s Chi-squared test
